# A novel bioassay to evaluate the potential of *Beauveria bassiana* strain NI8 and the insect growth regulator novaluron against *Lygus lineolaris* on a non-autoclaved solid artificial diet

**DOI:** 10.1093/jis/14.1.115

**Published:** 2014-09-01

**Authors:** Maribel Portilla, Gordon Snodgrass, Randall Luttrell, Stefan Jaronski

**Affiliations:** U.S. Department of Agriculture, Agricultural Research Service, Stoneville, MS 38732

**Keywords:** bioassay, biocontrol

## Abstract

A non-autoclaved solid diet was used to evaluate the entomopathogenic fungus *Beauveria bassiana* (Balsamo) Vuillemin (Hypocreales: Clavicipitaceae) strain NI8 and the insect growth regulator novaluron (Diamond® 0.83EC insecticide) for control of the tarnished plant bug, *Lygus lineolaris* (Palisot de Beauvois) (Hemiptera: Miridae). The diet was composed of toasted wheat germ, ground lima bean meal, soy flour, yolk of chicken eggs, inhibitor, and agar. It was prepared in one step by blending the ingredients in boiling water. The diet was used to bioassay *L. lineolaris* from the second instar to the adult stage. Fourth and fifth instars and adults of *L. lineolaris* were more susceptible than second and third instars to infection by *B. bassiana* , whereas second, third, and fourth instars had higher mortality than fifth instars 10 days after exposure to novaluron. No effects on longevity were observed in adults treated with novaluron when compared with the control, but longevity was significantly different from that of adults exposed to *B. bassiana* . Adults of *L. lineolaris* were maintained for over a month without changing the diet. The non-autoclaved diet is semi-liquid before it cools, which facilitates the mechanics of diet packaging similar to food packaging or lepidopteran diet preparation. This solid artificial diet for *Lygus* bugs provides improved research capacity for studying the ecology and susceptibility of *Lygus* spp. to a number of different control agents, including beneficial organisms, insect pathogens, and insecticidal toxins being developed for transgenic technologies.

## Introduction

The tarnished plant bug, *Lygus lineolaris* (Palisot de Beauvois) (Hemiptera: Miridae), attacks a wide variety of economically important herbaceous plants, vegetable crops, commercial flowering plants, fruit trees, and nursery stock ( Kelton 1975 ). Half of the cultivated plant species grown in the U.S. are listed as host plants for tarnished plant bugs ( Capinera 2001 ). *Lygus lineolaris* is a common pest of cotton, *Gossypium hirsutum* L. (Malvales: Malvaceae), throughout the southern and southeastern areas of the U.S. Cotton Belt. Yield losses in cotton due to this pest vary temporally and spatially ( Leonard and Cook 2007 ). Snodgrass et al. (2011) mentioned that species of *Lygus* in the U.S. infested >3 million ha of cotton in 2006, resulting in a yield loss of >240,000 bales ($75 million based on a 218 kg bale and $1.43/kg). Across the mid-south states of Arkansas, Louisiana, and Mississippi from 1991 to 2005, tarnished plant bugs infested 77-99% of cotton acreage ( Leonard and Cook 2007 ).

In the Delta Region of Mississippi, the frequency of insecticide use against *L. lineolaris* has varied and increased during the last 15 years. The annual number of insecticide applications in this area from 1991 to 1993 was less than one, but in 2006 an estimated 95% of Delta cotton (327,267 ha) was infested with tarnished plant bugs and received on average more than three insecticide applications ( Snodgrass et al. 2009 ). The cost of these control strategies has increased 10-fold, from $5 million to greater than $50 million in a time period of 15 years. One of the primary factors for this change is the wide commercialization and adoption of transgenic Bt cotton, which reduced early-season insecticide use for control of lepidopteran pests. This allowed *L. lineolaris* and other hemipteran pest populations previously suppressed by insecticides used for lepidopteran pests to progressively increase worldwide ( Liu et al. 2010 ). Effective management of *L. lineolaris* in cotton is complicated due to the mobility of the insect, and control has been based largely on insecticides. In 1993, a population of *L. lineolaris* in the Mississippi Delta was found to be highly resistant to pyrethroid insecticides, with multiple resistance to some organophosphate and cyclodiene insecticides ( Snodgrass 1996 ). Since then, resistance to pyrethroid and organophosphate insecticides has become widespread throughout the mid-south ( Snodgrass 1996 , Snodgrass and Scott 2002 , Snodgrass et al. 2009 ).

Among the various alternative methods proposed to control *L. lineolaris,* the insect growth regulator novaluron (Diamond® 0.83EC insecticide) and the entomopathogenic fungus *Beauveria bassiana* (Balsamo) Vuillemin (Hypocreales: Clavicipitaceae) have been tested. Barkley and Ellsworth (2004) , Smith et al. (2004) , Lund et al. (2006) , and Barbour (2008) found that novaluron showed promise as a new management tool for plant bug nymphs. Lund et al. (2006) mentioned that the use of *B. bassiana* for control of *Lygus* spp. in cotton was studied for more than two decades. Some of the investigations showed disadvantages. Leland and Behle (2004 , 2005) found that *B. bassiana* was sensitive to high temperature and solar radiation, Noma and Strickler (1999 , 2000) cited low adult mortality, Lund et al. (2006) found that *L. lineolaris* nymphs were less vulnerable than adults, and Spurgeon (2010) concluded that use of *B. bassiana* as a rescue treatment against *Lygus* in cotton may not be effective. Other reports have had more encouraging results and advocated additional research for use of the fungus as an alternative *L. lineolaris* control measure. Snodgrass and Elzen (1994) found that *B. bassiana* was moderately effective in reducing *L. lineolaris* in cotton at a rate of 1.1 liter/ha (reducing nymphs and adults by 53.8 and 20.2%, respectively). Steinkraus and Tugwell (1997) observed higher susceptibility to *B. bassiana* strains with isolates of the fungus from *L. lineolaris* than with isolates from other sources. Most importantly, a number of different studies indicated that some *B. bassiana* strains can be 10 times more virulent than the commercial strain (GHA) used in early studies. This high virulence was determined based on LC50, infection, and conidia production. The most promising isolate for control of *L. lineolaris* in the Delta is called NI8 or TPB3 ( Leland 2005 ; Leland et al. 2005 ; McGuire et al. 2005 , 2006). This isolate was found naturally infecting *L. lineolaris* in the Mississippi Delta ( Leland and Snodgrass 2005 ). Fargues and Remaudiere (1977) , Velez et al. (1997) , and McGuire (2002) suggested that isolates obtained from the environment and host would be more effective than isolates from other sources in controlling the target pest.

Current approaches for the evaluation of *B. bassiana* and novaluron, and any other control option, for activity against *L. lineolaris* rely on field experiments and bioassays using green beans, broccoli, or other material, such as florist wet foam as food ( Leland and Snodgrass 2005 , Leland 2005 , Leland et al. 2005 , McGuire et al. 2006 ). Detailed life-table studies or quantitative estimates of the impact of control agents on *L. lineolaris* life history require a bioassay option to study the impact of prolonged exposure for weeks following contact with the control agent. This is difficult with plant tissues or florist wet foam, which must be replaced routinely over the period of the study. Our bioassay with *L. lineolaris* studied the impact of *B. bassiana* and novaluron on adults and nymphs. It was the first bioassay that evaluated control agents against *L. lineolaris* by using solid artificial diet throughout its life cycle.

## Materials and Methods

### Tarnished plant bug colony

The study was conducted at the U.S. Department of Agriculture (USDA) Agricultural Research Service (ARS) South Insect Management Research Unit (SIMRU) in Stoneville, MS, USA. Adults were from a colony established in 1998 ( Portilla et al. 2011 ) and maintained previously at the USDA-ARS Biological Control Production Research and Rearing Unit (BCPRRU) in Starkville, MS. The colony was reared according to methods described by Portilla et al. (2011) that allowed obtaining sufficient numbers of insects with specific and similar ages. Insects were held in environmental chambers with a photoperiod of 16:8 (L:D), a temperature of 27ºC (±1.5ºC), and a relative humidity (RH) of 55% (±10%). For fungal infection and growth inhibition assays, one-day-old second (2-I), third (3-I), fourth (4-I), and fifth instars (5-I) and two-day-old adults (A) of *L. lineolaris* were used.

### Diet preparation

The non-autoclaved solid diet consisted of 13 ingredients ( Table 1 ). The diet was made by mixing the weighed components and blending them in boiling water and yolk from chicken eggs for about 4 min. The final mix (5 mL of diet per cup) was poured into individual 37 mL plastic cups (T-125, Solo Cup Company, www.solocup.com ) and kept at room temperature to cool and solidify before use.

**Table 1.**
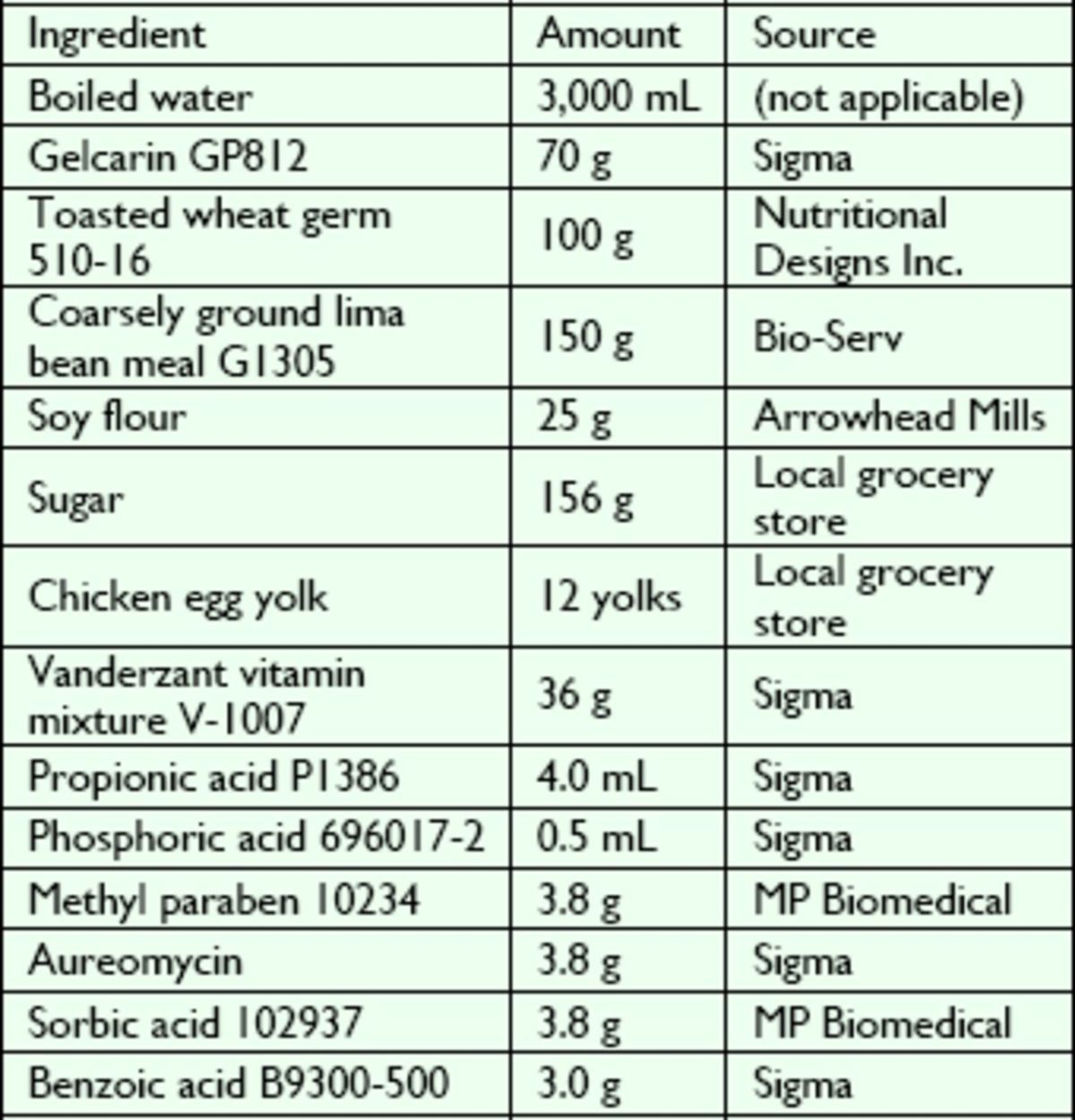
Diet components of the new non-autoclaved solid artificial diet for *L. lineolaris* (yield per batch: approximately 1 gallon of diet).

### Fungal isolate

The NI8 isolate of *B. bassiana* was obtained from the collection of the USDA-ARS-SIMRU and was produced in a biphasic cul ture system that simulated industrial-scale production according to the method described for solid-substrate fermentation of *B. bassiana* by Bradley et al. (2002) and Grace and Jaronski (2005) . To determine spore germination, harvested spores were examined for sporevi ability (percentage of germination) ( Velez et al. 1997 , Grace and Jaronski 2005 ). Spore concentrations (spores per mm ^2^ ) were quanti fied by counting spores deposited on five dis- disposable microscope cover slips of 2.2 cm ^2^ (S17525, Fisher Scientific, www.fishersci.com ). The spray was done by using a specially designed spray tower modified from a Burgerjon tower ( Burgerjon 1956 ). Cover slips were placed at five locations equally spaced from center to quadrants of a filter-paper-lined Petri dish (15 cm diam × 3 cm depth; 170C, Pioneer Plastics, www.pioneerplastics.com ) that was used to spray the insects. After the spray treatment, the Petri dish with cover slips was removed from the spray tower, and each cover slip was placed into a separate vial that contained 2 mL 0.04% Tween 80. The vials were vortexed for about 1 min to wash the spores from the cover slip into the solution. Numbers of spores suspended in solution from each vial were deter-determined by using a hemacytometer ( Velez et al. 1997 , Grace and Jaronski 2005 ). This process was repeated six times, and the numbers of spores per mm ^2^ were analyzed with analysis of variance (ANOVA). Means per location were separated with the Tukey HSD test ( *P* = 0.05). Numbers of spores applied were corrected for viability. Harvested spore powder (0.5 g) containing 7.6 × 10 ^10^ spores per gram was suspended in 50 mL 0.04% Sylwet L77 and diluted to obtain a final concentration of 1.5 × 10 ^9^ spores per mL. Aliquots of 6 mL of this suspension were sufficient to obtain a desired concentration of about 500 viable spores per mm ^2^ to inoculate the *L. lineolaris* stages, a concentration that was similar to that used by Ugine (2011) .

### Bioassay procedure

A single-concentration screening assay was conducted to evaluate longevity, mortality, infection, and growth inhibition of *L. lineolaris* treated as nymphs or adults. Twenty groups of 10 insects (four replicate groups of one- to two-day-old 2-I, 3-I, 4-I, and 5-I, and of two-day-old male and female [at equal ratio] adults) were used per treatment (control, fungus, and insecticide). The groups were sprayed with 6 mL of water (control), 6 mL of *B. bassiana* strain NI8 suspension at 492 ± 71 spores per mm ^2^ (fungus), or 6 mL of the growth regulator Diamond 0.83EC solution (1.44 mL of Diamond 0.83EC [99.6 mg of novaluron per mL] in 50 mL 0.04% Sylwet L77 water solution). All treatments were applied with the specially-designed spray tower modified from a Burgerjon tower. After application, adults and nymphs were released in an insect observation cage (1466A, BioQuip, www.bioquip.com ) to let them dry and then transferred individually into a cup with solid diet. Adults and nymphs were examined daily for mortality and for molting (nymphs). Insects sprayed with *B. bassiana* that molted were transferred to a new cup to avoid contact with the infected exuviae. Dead insects were kept in the same cup and were checked daily for sporulation. Adults and nymphs were held in an environmental room at 27ºC, 65% RH, and a photoperiod of 12:12 (L:D). Insects were kept until all were dead.

**Table 2.**
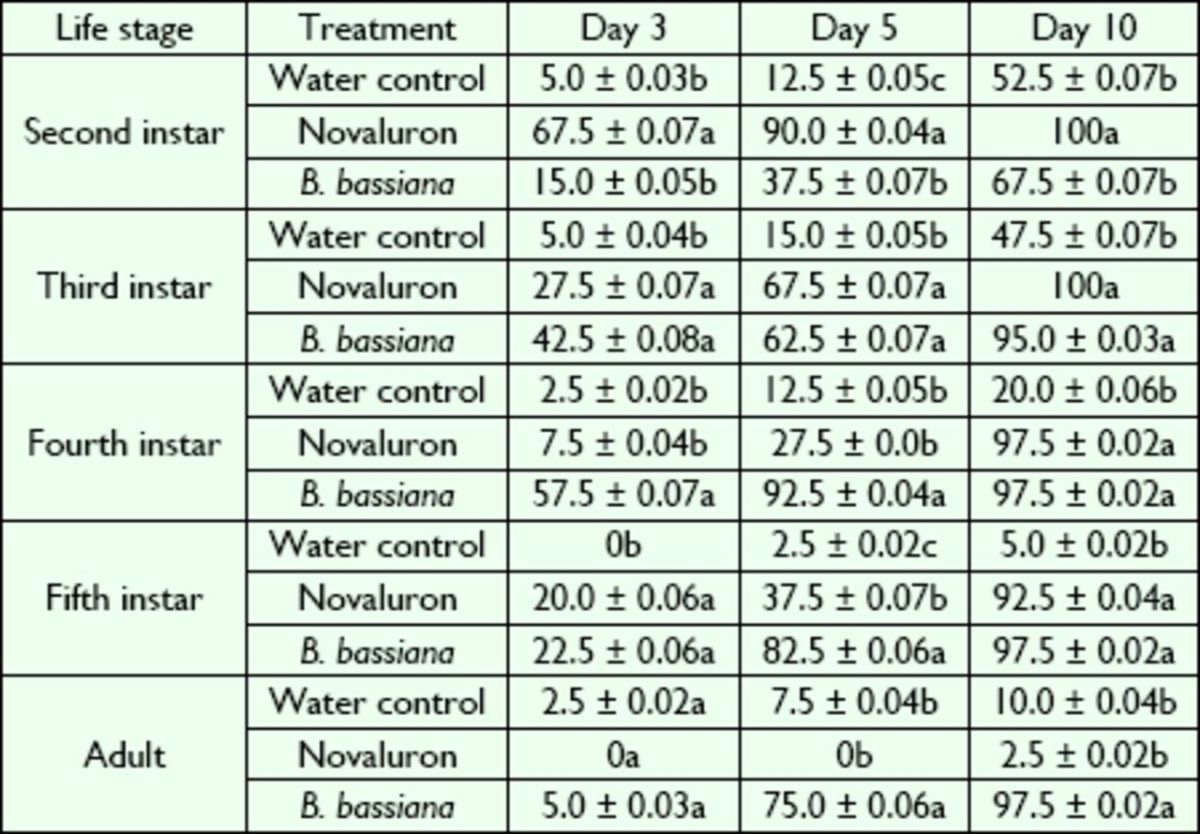
Mean (± SE) percentage of mortality in *L. lineolaris* fed solid *Lygus* diet and exposed to *B. bassiana* or the insect growth regulator novaluron

Means per life stage within a column followed by a different letter were significantly different at *P* ≤ 0.05 (Tukey test).

### Statistical analysis

The experiment was set up as a completely randomized design with a factorial arrangement of 3 × 5 × 3 for mortality and 3 × 5 for longevity and molt (three treatments: water [control], *B. bassiana* , and novaluron; five stages of *L. lineolaris* ; and three evaluation times: Day 3, Day 5, and Day 10). Each treatment combination was repeated four times. Statistics were performed using SAS system software (SAS Institute, www.sas.com ). Nonparametric estimates of the survival functions of *L. lineolaris* stages were compared between treatments by using the PROC LIFETEST procedure of SAS. The analyses controlled for repetitions of the experiment by using the strata statement, and insect development was included as a covari-ate in the test statement ( Allison 1995 ). Statistical differences in the survival of *L. lineolaris* stages between the treatments were declared based on the log-rank statistic. Mortality, longevity, fungal infection, sporulation, and molt were analyzed by using the PROC GLM procedure to detect differences between treatments.

## Results

### Age-dependent mortality of *L. lineolaris*

Novaluron had a highly significant effect on mortality of 2-I of *L. lineolaris* at all evaluation times, Day 3 ( *F* = 33.44; df = 2, 17; *P* < 0.01), Day 5 ( *F* = 42.20; df = 2, 17; *P* < 0.01), and Day 10 ( *F* = 14.72; df = 2, 17; *P* < 0.01), when compared with the water control and *B. bassiana* treatment ( Table 2 ). Water-treated and *B. bassiana* -treated 2-I did not differ in mortality at Day 3 and Day 10 ( Table 2 ). The 3-I treated with water had greater survival than those treated with novaluron and *B. bassiana* ( Table 2 ). Mortality of 4-I was greater for *B. bassiana* at Day 3 and Day 5 but similar to novaluron at Day 10 ( Table 2 ). Mortality of 5-I was similar for novaluron and *B. bassiana* at Day 3 and Day 10 ( Table 2 ). At Day 5, mortality of *B. bassiana* –treated 5-I was greater than that of novaluron-treated 5-I ( Table 2 ). Adult mortality was significantly higher in the *B. bassiana* treatment at Day 5 ( *F* = 77.72; df = 2, 17; *P* < 0.01) and Day 10 ( *F* = 235.23; df = 2, 17; *P* < 0.01) than in the water control and the novaluron treatment.

**Table 3.**
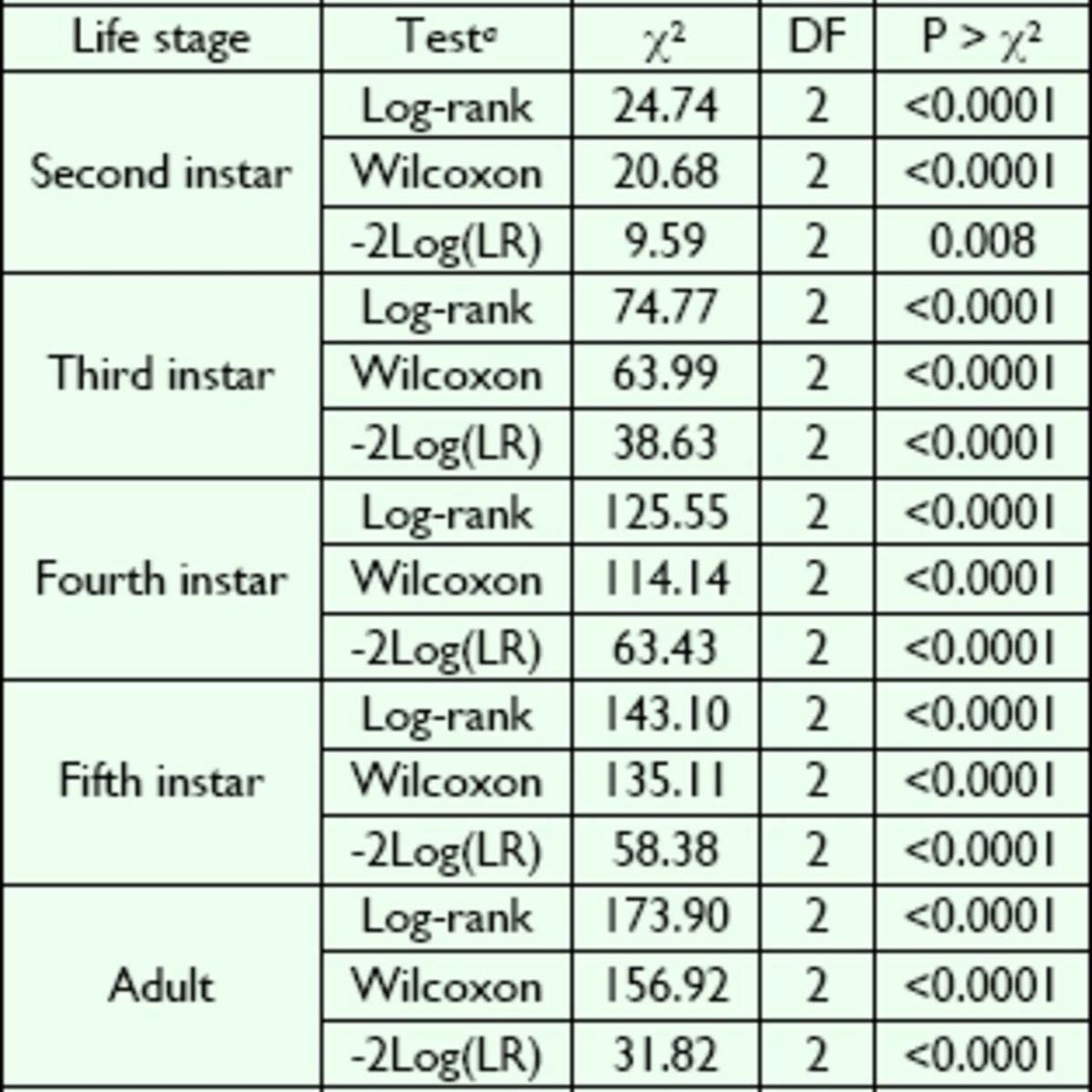
Test of equality with the strata statement in PROC LIFETEST for *L. lineolaris* fed solid *Lygus* diet and exposed to *B. bassiana* or novaluron.

Novaluron had no measurable activity against adults. Adult mortality did not show significant differences between treatments at Day 3 *(F* = 1.02; df = 2, 17; *P* = 0.364) or between water and novaluron at Day 5 and Day 10 ( Table 2 ).

### Longevity and growth inhibition

**Figure 1.**
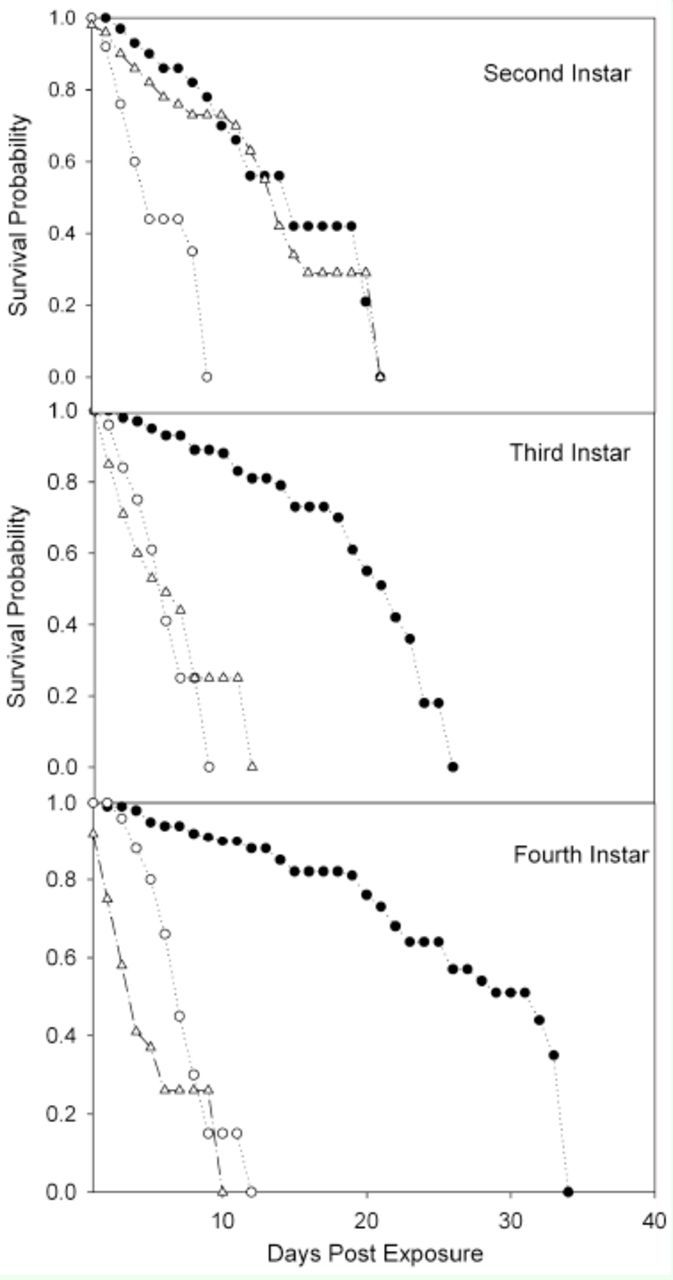
Survival of early stages of *Lygus Lineolaris* (fed solid artificial diet) after spray exposure to the entomopathogenic fungus *Beauveria bassiana* (white triangles) or the insect growth regulator nuvaluron (white circles). Controls (black circles) were sprayed with water. High quality figures are available online.

**Figure 2.**
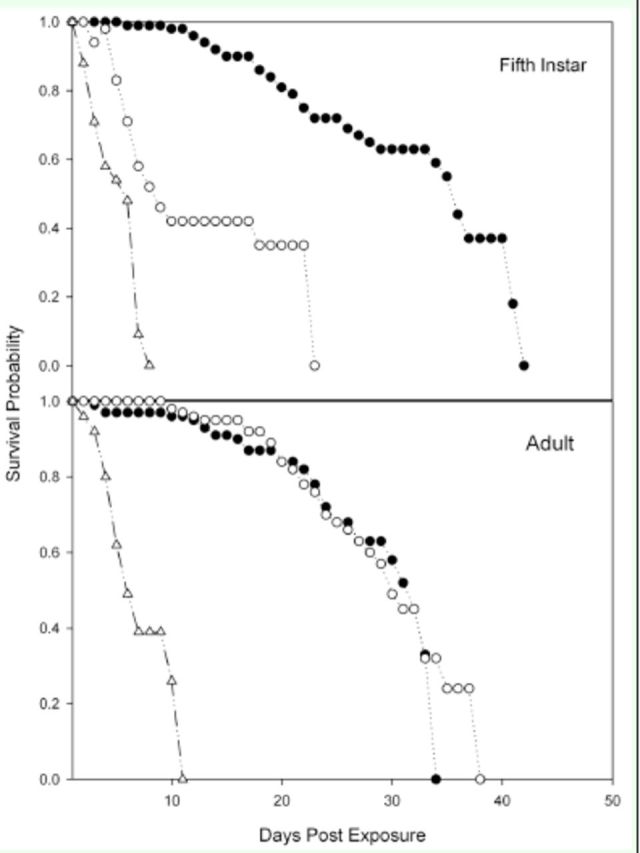
Survival of late stages of *Lygus lineolaris* (fed solid artificial diet) after exposure to the entomopathogenic fungus *Beauveria bassiana* (white triangles) and the insect growth regulator novaluron (white circles). Controls (black circles) were sprayed with water. High quality figures are available online.

Survival rates for each combination of treatments and *L. lineolaris* stages are presented in Figures 1 and 2 . The log-rank and Wilcoxon tests for homogeneity indicated significant differences between treatments in each *L. lineolaris* stage when compared with the water control ( Table 3 ). Figure 1 showed that 2-I were more likely to survive after fungus application, whereas 5-I were more likely to survive after novaluron application ( Figure 2 ). Table 4 showed a longer mean longevity in the water control for all *L. lineolaris* stages except for adults, where no significant differences were found, when compared with novaluron. No significant differences in longevity were observed between novaluron and *B. bassiana* treatments for 3-I, 4-I, and 5-I ( Table 4 ). Growth inhibition was determined by percentage of molt, and this percentage was highest in all *L. lineolaris* immature stages sprayed with water (control), followed by the insects sprayed with *B. bassiana* ( Table 4 ). Percentage of molt was highly reduced in all immature stages treated with novaluron ( Table 4 ).

**Table 4.**
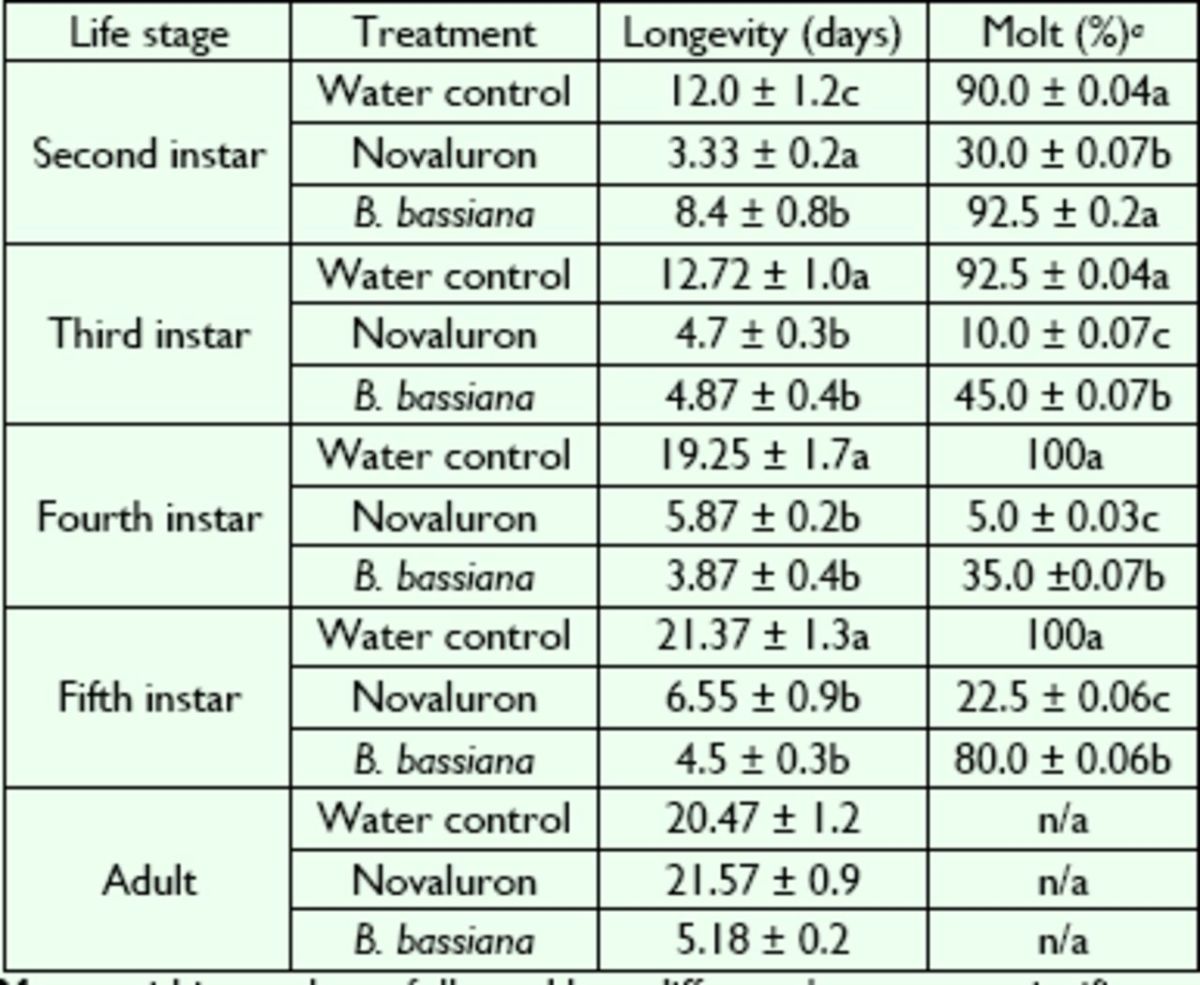
Mean (± SE) longevity and growth inhibition in *L. lineolaris* fed solid *Lygus* diet and exposed to *B. bassiana* or novaluron.

Means within a column followed by a different letter were significantly different at *P* ^ 0.05 (Tukey test). 1 *a* n/a, not applicable.

**Table 5.**
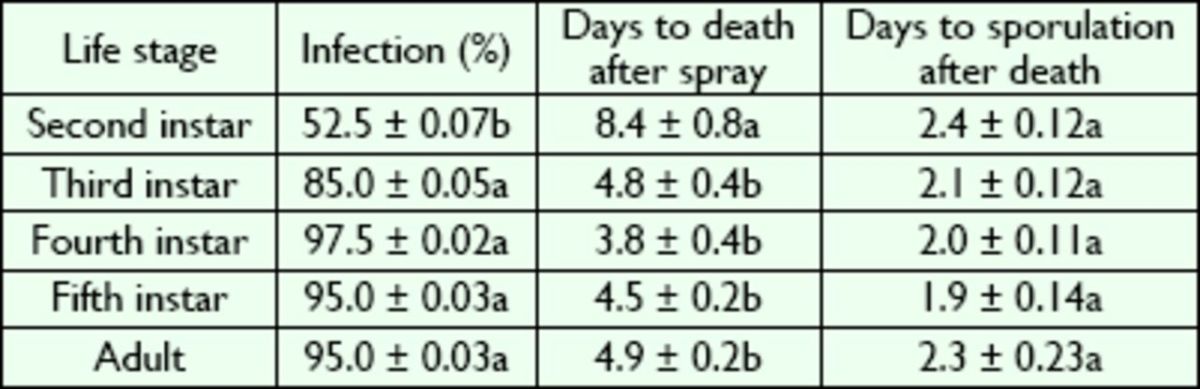
Mean (± SE) percentage of infection and time to death and sporulation in *L. lineolaris* sprayed with *B. bassiana* and held on a solid *Lygus* diet.

Means within a column followed by a different letter were significantly different at *P* < 0.05 (Tukey test).

### Infection by *B. bassiana* and percentage of sporulation

The pathogenicity of *B. bassiana* observed in the bioassayed *L. lineolaris* held on solid *Lygus* diet is shown in Table 5 . The percentage of infection of 2-I (on average 52.5%) was significantly lower than that of the rest of the *L. lineolaris* stages *(F =* 13.38; df = 2, 195; *P* < 0.01), and it took about two times longer for 2-I than for later instars and adults to die ( Table 5 ). No significant differences were found in infection rates and days to death between 3-I, 4-I, 5-I, and adults ( Table 5 ). Infection rates and days to death ranged from 85 to 97% and 3.8 to 4.9 days, respectively. No significant differences between stages occurred in days to sporulation *(F =* 13.38; df = 2, 195; *P >* 0.01), and these values ranged from 1.9 to 2.3 days after the insects’ deaths ( Table 5 ).

## Discussion

The significant differences in mortality, longevity, fungal infection, growth inhibition, and sporulation obtained in this study indicated that the novel bioassay for *L. lineolaris* on solid *Lygus* diet was effective in determining the activity of *B. bassiana* and novaluron against all developmental stages of the tarnished plant bug. Our data ( Tables 4 and 5 ) confirmed that fungal sporulation and growth disruption in *L. lineolaris* held on solid artificial diet at 27°C occurred in a period of time that ranged from two to 10 days when a concentration of 492 ± 71 spores per mm2 of *B. bassiana* was used. The fungal incubation period (i.e., the sum of days to death and days to sporulation) ranged from 5.8 to 10.8 days, depending on insect developmental time ( Table 5 ). These results were comparable with those from previous laboratory studies that reported high mortality for *Lygus* spp. At three days or longer after inoculation at 28°C ( Leland et al. 2005 ; McGuire et al. 2005 , 2006; Spurgeon 2010 ). Determining the time needed for pesticides to work is important in conducting a bioassay. This time period can be more than 20 days at low temperature (12.8°C) and low concentration (1 × 106 conidia per mL) ( Spurgeon 2010 ) and may affect control mortality due to excessive insect handing when feeding the insects. For example, Lund et al. (2006) reported a control mortality of 82.8% for *L. lineolaris* nymphs and 56.4% for adults. The experimental results summarized in our study demonstrated that insects bioassayed on solid *Lygus* diet had low mortality in the control. The solid diet is used just one time from the day of insect inoculation until the end of the bioassay. This avoids the three times weekly food changes that are commonly required in the standard method when fresh green beans or florist wet foam are provided to the remaining alive insects until the end of the assay ( Steinkraus and Tugwell 1997 ; Liu et al. 2002 ; Leland et al. 2005 ; McGuire et al. 2005 , 2006; Spurgeon 2010 ). Avoiding insect handling could also minimize contaminateon. In our study, there was no mortality in the control due to fungal growth either by *B. bassiana* or other fungal contaminants. The inhibitors can last for about 20 days; after that, *B. bassiana* from infected insects can slowly grow on the diet. Figures 1 and 2 shows the time that nymphs and adults of *L. lineolaris* were kept in the solid-diet cups to obtain longevity estimations. The shortest longevity in the controls was obtained in 2-I and 3-I and indicated that the diet did not work well for early immature stages. However, the mortality for 2-I and 3-I in this study was still two-fold lower when compared with the green-bean technique (82.4% at 10 days after application) ( Lund et al. 2006 ). The high mortality in early instars suggested that the diet cannot be used for life cycle studies; however, it worked well in our bioassay for late-instar nymphs and adults of *L. lineolaris* . The survival trend for all *L. lineolaris* stages was significantly different between treatments ( *P* = 0.01 for log-rank test and Wilcoxon test) ( Table 3 ). No bioassays were used to compare total adult longevity In the control vs. treated insects. However, the adult longevity obtained in this experiment for 4-I (on average 19.2 days) and 5-I (21.0 days) that reached adulthood and for adults (21.6 days) ( Table 4 ) fit the longevity range found by Ugine (2012) . He found that *L. lineolaris* longevity ranged between 17.0 and 39.4 days at temperatures lower than 32°C when insects were reared on green beans. The probability of survival presented in this investigation indicated that all *L. lineolaris* stages can survive long enough on the solid *Lygus* diet to measure growth disruption and the life cycle of *B bassiana* on treated insects including the pathogenesis and sporogenesis phases for *B bassiana* .

Most of the mortality studies on *L. lineolaris* are based on field and laboratory populations of adults and nymphs of unknown ages. Our investigation classified mortality from early nymphal to adult stages. The cumulative mortality of *L. lineolaris* obtained in Table 1 showed that early-instar nymphs were more susceptible to novaluron, whereas late-instar nymphs were more susceptible to *B. bassiana.* The highest initial mortality of more than 65% occurred in 2-I treated with novaluron at Day 3, and all novaluron-treated 2-I were dead by Day 10. Late-instar nymphs were found to have a lower initial mortality response to novaluron, but mortality increased at Day 10 to 100% for 3-I and over 92% for 4-I and 5-I. Mortality of immature stages was different with *B. bassiana.* No significant differences were found between 2-I treated with *B. bassiana* and those in the water control (15.0 and 5.0%, respectively) at Day 3, and although the mortality of *B. bassiana-treated* 2-I increased at Day 5, more than 30% of the population survived the application at Day 10. The high mortality (greater than 47%) obtained in the water control at Day 10 for 2-I and 3-I indicated, as discussed before, that these instars had a low acceptance to the diet; therefore, the percentage of survival could be much higher for early-instar nymphs treated with *B. bassiana* under field conditions. Second instars of *L. lineolaris* were less susceptible to fungal infection than 3-I, 4-I, and 5-I. The highest initial (Day 3) mortality was found for 3-I and 4-I at 42 and 57%, respectively. All instars treated with *B. bassiana* except for 2-I ended with a mortality of 95% or more. These data suggested that *B. bassiana* and novaluron can cause high initial mortality in *L. lineolaris,* but also that a population of early instars may survive *B. bassiana* application. These results are comparable to those reported by Liu et al. (2002) , who found that mortality in second instars of *L. lineolaris* varied from 35 to 98% among treatments with 18 *B. bassiana* isolates. No data were presented for late nymphal instars. Similar results were obtained by Lund et al. (2006) , who reported initial mortality of 22.3% at Day 2 after novaluron spray, increasing to 66.9% at Day 5 and 97.1% at Day 10. In their study, treatments with *B. bassiana* showed a similar result, with an initial mortal-Journal of Insectity of 25.5% at Day 2 and a final mortality of 95.4% at Day 10. No ages or instars of the nymphal stages were mentioned. Our results showed that under laboratory conditions, late-instar nymphs of *L. lineolaris* were highly susceptible to *B. bassiana* and percentage of infection did not differ statistically from that of adults. Previous studies ( Leland 2005 , Leland et al. 2005 , McGuire et al. 2006 ) have demonstrated that isolate NI8 had higher sporulation than other isolates. Leland (2005) estimated an SC50 (S: sporulation) 13.6-fold higher than that of the commercial strain. In our study, novaluron did not affect adults, and the initial and final mortality did not differ statistically from that in the water control. In novaluron-treated nymphs, percentage of molt varied depending on insect development time, but by Day 10 did not affect the percentage of immature mortality that ranged from 93 to 100%.

Field studies have shown very low susceptibility to *B. bassiana* in *Lygus* nymphs ( McGuire 2002 , Lund et al. 2006 , Gonzáles-Santarosa et al. 2010 ) based on sampled populations in the field at 5, 10, or 14 days after treatment. The estimated population data after those time periods could have been skewed because the collected nymphs used for that estimation may have been eggs or first or second instars at the time of the application. A similar situation may have occurred for adults, which may have originated from treated late-instar nymphs. Therefore, estimates of nymphal and adult populations in the field with insects of unknown ages could produce variation in mortality estimates. For example, Snodgrass and Elzen (1994) reported a reduction in nymphal population of 53.8%, whereas McGuire et al. (2006) found a reduction in the nymphal population of less than 10% at 10 and 14 days after treatment. The McGuire study mentioned that the nymphs in the study were probably eggs at the time of application. In the case of adult populations, studies that reported reductions in adult populations under field conditions may have indicated that *B. bassiana* was suppressing adults and late instars.

*Beauveria bassiana* and novaluron highly affected *L. lineolaris* survival when they were applied directly to the insects in our test. Both products could be considered to have good potential to control *L. lineolaris;* however, under laboratory conditions, the low susceptibility of early-instar nymphs to *B. bassiana* and the lack of effect of novaluron on adults reduce their effectiveness for *L. lineolaris* control. As suggested by Lund et al. (2006) , the combination of both products could greatly increase mortality. The authors found greater initial mortality at Day 2 and Day 5 in the combined treatments compared with *B. bassiana* and novaluron alone; however, at Day 10, the mortality in combined and individual treatments did not differ.

The solid artificial diet for *Lygus* bugs, although not optimal for early immature stages, provides a useful tool for future laboratory studies with *Lygus* spp. Use of this diet will facilitate the testing and evaluation of biological control agents before conduction of field experiments.
